# Risk factor analysis of the decrease in gait speed among Japanese older outpatients with polypharmacy

**DOI:** 10.1186/s40780-019-0152-4

**Published:** 2019-11-16

**Authors:** Masataka Deguchi, Keigo Nishida, Tomoyuki Enokiya, Kazuya Ooi

**Affiliations:** 1 Life Pharmacy, 475-1, Kozubeta, Tsu, Mie 514-0061 Japan; 2 Laboratory of Clinical Pharmacology, Graduate School of Pharmaceutical Sciences, Suzuka University of Medical Science, 3500-3 Minamitamagaki, Suzuka, Mie 513-8670 Japan; 3 Laboratory of Immune Regulation, Graduate School of Pharmaceutical Sciences, Suzuka University of Medical Science, 3500-3 Minamitamagaki, Suzuka, Mie 513-8670 Japan; 4 Labolatory of Pharmacoinformatics, Department of Pharmaceutical Sciences, Suzuka University of Medical Science, 3500-3 Minamitamagaki, Suzuka, Mie 513-8670 Japan

**Keywords:** Older outpatients, Polypharmacy, Gait speed, Prescription contents, Calcium channel blocker, Stratum corneum moisture content

## Abstract

**Background:**

Both polypharmacy and frailty are critical issues faced by the elderly. The decrease in gait speed is an index of frailty, and it is generally associated with falls and fractures, which are risk factors requiring the need for support or long-term patient care. In this study, we assess the risk factors responsible for the decrease in gait speed in older outpatients with polypharmacy.

**Methods:**

Thirty-one persons (13 men, 18 women) aged 65 years or above and regularly taking 5 or more internal medications participated in this study.

**Results:**

Propensity score-adjusted multivariate logistic analysis showed that only number of medications was associated with the risk of decreasing gait speed (odds ratio: 16.00, 95% confidence interval:1.72–149.00, *p* value = 0.0149). A negative correlation was found between the number of medications and gait speed. In addition, the gait speed of the calcium channel blocker medication group was significantly slower than that of the non-medication group.

**Conclusion:**

These results suggest that not only the number of medications but also the prescription contents is a risk factor for decrease in gait speed and may serve as indexes to identify patients at high risk of requiring support or long-term care.

## Background

The percentage of elderly in the Japanese population was estimated to be 28.3% by April 2019 [1]. Accordingly, the number of older outpatients has remarkably risen owing to the rapid aging of the population as well as the increase in chronic medical conditions such as hypertension, diabetes mellitus, and dyslipidemia that are often accompanied by multiple diseases, resulting in polypharmacy [2]. Polypharmacy leads to not only decreases in patient compliance but also increases in health care costs [3]. Moreover, it has been reported that the concomitant assumption of more than 5 or 6 medications can lead to risk factors such as falling [4] or adverse drug reactions [5], respectively.

Besides polypharmacy, Fried et al. proposed the concept of frailty as an additional issue in the elderly [6]. In recent years, the importance of frailty has attracted widespread interest to prevent long-term care owing to its pathophysiology and diagnosis [7]. The authors proposed 5 symptoms as the phenotype of frailty: 1) weight loss, 2) weakness, 3) exhaustion, 4) slowness, and 5) low activity. In addition, they suggested that if patients showed more than three of these symptoms, they should be deemed as frailty [6]. Other studies have been reported that decreases in gait speed, which is an index of slowness, is associated with falling and fractures [8–11]. For the elderly, these are risk factors requiring the need for support or long-term patient care [12]. Therefore, it can be assumed that the evaluation of the gait speed at the community pharmacy settings might lead to health care support for patients.

By identifying a risk factor associated with the decrease in gait speed, it is possible to detect in advance a patient with a high risk of frailty, falls, and fracture. The aim of this study is to assess the risk factors for the decrease in gait speed in older outpatients with polypharmacy.

## Methods

### Patient selection

Between November 2016 and April 2018, 65 years or older outpatients who visited the Heart Pharmacy Zaitaku Center (Matsusaka-city, Mie-Pref.) and regularly took 5 or more internal medications, were enrolled in the study. Patients with gait disturbance were excluded from the study.

### Propensity score-adjusted multivariate logistic analysis

Risk factors for the decrease in gait speed were evaluated by performing a multivariate logistic analysis with an adjusted propensity score. When explaining the instructions indicated on a medication at our pharmacy, we performed a multifaceted listening to older outpatients who appeared to have issues with walking. A tendency of these patients to complain about itchy dry skin was observed. Furthermore, it was reported that a low body mass was associated with increased dry skin manifestations and decreased skin elasticity in community-dwelling older adults [13], thus suggesting that dry skin may be an indicator of frailty. Therefore, in the present study, we selected the stratum corneum moisture content, an indicator of dry skin, as a risk factor candidate. Overall, this analysis identified 8 risk factors for frailty: sex, height, weight, thigh circumference, gait speed, body mass index (BMI), stratum corneum moisture content, and number of medications. Height (cm), weight (kg), and thigh circumference (cm) were measured, and their BMI was calculated using the following formula: BMI = Weight (kg) / [Height (m)]^2^.

The participants were asked to walk 5 m in the pharmacy room at their usual pace, and then their gait speed (m/s) was calculated. The stratum corneum moisture content was measured non-invasively in the forearm of the patients with a portable skin moisture meter (Courage + Khazaka, Germany; HP10-N) by using the electric capacitance method. The resulting measurements were indicated using a relative value ranging from 0 to 99 arbitrary unit (a.u.). The environment of the room where the measurements were conducted was independent of the outside air, and the room temperature was kept within the range between 15 and 25 °C by using an air conditioner to remove the effect of perspiration. After acclimatization by leaving the forearm exposed from the clothes and standing still for about 15 min, the measurement was performed 3 times for each patient, and the mean value was calculated. The number of medications was calculated by adding the number of internal drug prescription filled for patients of the Heart Pharmacy Zaitaku Center and all other medical institutions, and it was confirmed via a medication record kept by a pharmacist. The medications to be counted were those that have been taken continuously for more than 1 month. This calculation was performed at the time when the gait speed and stratum corneum moisture content were measured. Based on the therapeutic category number [14] of the medicine that each patient was taking, we tabulated and analyzed the number of patients for each therapeutic category.

The participants were divided based on the J-CHS standards, which are the criteria used in the diagnosis of frailty in Japan, into the following two groups: 1) fast gait speed group with a gait speed of ≥1.0 m/s and 2) slow gait speed group with a gait speed of < 1.0 m/s. The prescription contents for both the fast and slow gait speed group of patients were tabulated. Subsequently, the difference in the proportion of patients belonging to each group was analyzed. Furthermore, the difference in gait speed between the medication and non-medication group for medicines of the therapeutic category with differences in the number of patients was analyzed.

### Statistical analysis

The propensity scores for a target variable were calculated based on a multivariate logistic model using the 7 other variables. For quantitative variables (age, number of medications, stratum corneum moisture content, height, weight, BMI, and thigh circumference), each cut-off value was set by the receiver operating characteristic curve analysis.

The correlation between the number of medications and gait speed was analyzed using the Spearman’s rank correlation coefficient. The differences in variables between the two groups were analyzed using the Mann Whitney U test as well as the Fisher’s exact test.

All statistical analyses were performed using EZR (Saitama Medical Center, Jichi Medical University, Saitama, Japan, version 1.33), which is a graphical user interface for R (the R Foundation for Statistical Computing, Vienna, Austria, version 3.3.1). More precisely, it is a modified version of the R commander (version 2.3–0) designed to add statistical functions frequently used in biostatistics [15]. The significance was established when the *p* value was < 0.05.

### Heat map analysis

Using Microsoft Excel with the vertical and horizontal axis as patient and therapeutic category number, respectively, a heat map was created by coloring the cells with the therapeutic category number of the medicine that each patient was taking. The trends were visually examined by sorting the heat map based on the gait speed of each patient.

### Ethical consideration

This study was approved by the institutional review board of the Suzuka University of Medical Science (Approval No.274, September 5, 2016).

## Results

### Participants and propensity score-adjusted multivariate logistic analysis

**Fig. 1:**
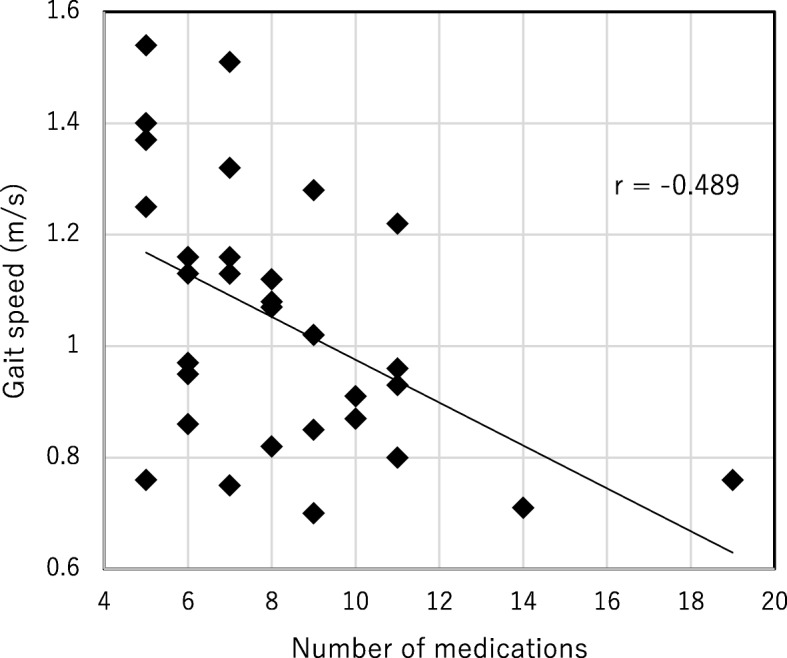
Correlation between number of medications and gait speed. Examined by the Spearman’s correlation coefficient

A total of 31 participants (13 men, 18 women, 79.00 [65.00–89.00] years) was enrolled in the study (Table 1). Propensity score-adjusted multivariate logistic analysis showed that only number of medications was associated with the risk of decreasing gait speed (odds ratio [OR]: 16.00, 95% confidence interval (CI):1.72–149.00, *p* value = 0.0149). In contrast, stratum corneum moisture content was not significantly associated with the risk of decreasing gait speed (odds ratio [OR]: 0.362, 95% confidence interval (CI):0.07–1.88, *p* value = 0.227) (Table 2). Height, weight and BMI were not analyzed due to lack of freedom. In addition, a negative correlation between the gait speed and number of medications was found (Fig. 1).

**Table 1:** Patient demographic characteristics (*n* = 31)

Characteristics	Number (%) or Median [range]	
Men	13 (41.93)	
Age	79.00	[65.00–89.00]
Gait speed (m/s)	1.02	[0.70–1.54]
Number of medications	8.00	[5.00–19.00]
Stratum corneum moisture content (a.u.)	33.33	[17.00–49.67]
Height (cm)	154.00	[140.00–169.00]
Weight (kg)	53.00	[40.00–75.00]
Thigh circumference (cm)	43.00	[36.00–51.00]
BMI	22.52	[16.44–27.99]

**Table 2:** Propensity score-adjusted multivariate logistic analysis (n = 31)

Risk factors	OR (95% CI)	C statistics	*p* value
Sex	4.91 (0.06–402.00)	0.996	0.479
Age	4.73 (0.91–24.50)	0.684	0.0643
Number of medications	16.00 (1.72–149.00)	0.794	0.0149
Stratum corneum moisture content (a.u.)	0.362 (0.07–1.88)	0.756	0.227
Height (cm)	no data		
Weight (kg)	no data		
BMI	no data		
Thigh circumference (cm)	0.911 (0.04–22.70)	0.982	0.955

### Relationship between the gait speed and prescription contents

As a result of a further analysis, it was found that the proportion of the slow gait speed group patients on 117.Psychotropic agents, 217.Vasodilators, and 333.Anticoagulant tended to be higher (Table 3), therefore we focused among this group on 217.Vasodilators which the group with the largest number of people in the above three groups.

**Table 3:** Number of patients aggregated based on therapeutic category medication

Therapeutic category number	Name of therapeutic category	All patients n = 31		Fast gait speed group *n* = 16		Slow gait speed group *n* = 15		*p* value
112	Hypnotics and sedatives, antianxietics	11	35.48%	5	31.25%	6	40.00%	0.716
113	Antiepileptics	1	3.23%	0	0.00%	1	6.67%	0.484
114	Antipyretics, analgesics and anti-inflammatory agents	3	9.38%	1	5.88%	2	13.33%	0.600
116	Antiparkinsonism agents	1	3.13%	0	0.00%	1	6.67%	0.484
117	Psycotropic agents	3	9.68%	0	0.00%	3	20.00%	0.101
119	Other agents affecting the central nervous system	2	6.45%	0	0.00%	2	13.33%	0.226
124	Antispasmodics	1	3.23%	0	0.00%	1	6.67%	0.484
133	Antimotionsickness agents	1	3.23%	0	0.00%	1	6.67%	0.484
212	Antiarrhythmic agents	3	9.68%	1	6.25%	2	13.33%	0.600
213	Diuretics	2	6.45%	1	6.25%	1	6.67%	1.000
214	Antihypertensives	24	77.42%	14	87.50%	10	66.67%	0.220
217	Vasodilators	20	64.52%	8	50.00%	12	80.00%	0.135
218	Agents for hyperlipidemias	22	70.97%	12	75.00%	10	66.67%	0.704
219	Other cardiovascular agents	7	22.58%	2	12.50%	5	33.33%	0.220
223	Expectorants	3	9.68%	1	6.25%	2	13.33%	0.600
231	Antidiarrheals, intestinal regulators	2	6.45%	1	6.25%	1	6.67%	1.000
232	Agents for peptic ulcer	22	70.97%	10	62.50%	12	80.00%	0.433
233	Stomachics and digestives	2	6.45%	1	6.25%	1	6.67%	1.000
234	Antiacids	5	16.13%	3	18.75%	2	13.33%	1.000
235	Purgatives and clysters	5	16.13%	1	6.25%	4	26.67%	0.172
239	Other agents affecting digestive organs	3	9.68%	1	6.25%	2	13.33%	0.600
259	Other agents for uro-genital and anal organs	2	6.45%	0	0.00%	2	13.33%	0.226
311	Vitamin A, D and preparations	6	19.35%	3	18.75%	3	20.00%	1.000
313	Vitamin B preparations (except Vitamin B1)	3	9.68%	2	12.50%	1	6.67%	1.000
317	Mixed vitamin preparations (except mixed vitamin preparations compounded of vitamin A and D)	2	6.45%	2	12.50%	0	0.00%	0.484
321	Calcium compounds and preparations	1	3.23%	0	0.00%	1	6.67%	0.484
322	Mineral preparations	1	3.23%	0	0.00%	1	6.67%	0.484
333	Anticoagulants	6	19.35%	1	6.25%	5	33.33%	0.083
339	Other agents relating to blood and body fluides	18	58.06%	8	50.00%	10	66.67%	0.473
392	Antidotes	1	3.23%	1	6.25%	0	0.00%	1.000
394	Agents for treatment of goat	5	16.13%	3	18.75%	2	13.33%	1.000
396	Antidiabetic agents	9	29.03%	6	37.50%	3	20.00%	0.433
399	Agents affecting metabolism, n.e.c.	5	16.13%	3	18.75%	2	13.33%	1.000
449	Other antiallergic agents	4	12.90%	2	12.50%	2	13.33%	1.000
520	Chinese medicines	1	3.23%	1	6.25%	0	0.00%	1.000
614	Antibiotic preparations acting mainly on gram-positive bacteria and mycoplasma	2	6.45%	0	0.00%	2	13.33%	0.226
622	Anti-tuberculous agents	1	3.23%	0	0.00%	1	6.67%	0.484
624	Synthetic antibacterials	1	3.23%	0	0.00%	1	6.67%	0.484

*p* value based upon Fisher’s exact test

In this therapeutic category, medicines classified as 217.Vasodilators were mainly calcium channel blocker (CCB) such as amlodipine, nifedipine, benidipine, and nitrates. However, the CCB cilnidipine was classified as 214.Antihypertensives. Therefore, the patients were divided into three groups according to the medicines they took, i.e., CCB, other vasodilators (Remains after removing CCB from vasodilators), and other antihypertensives (Remains after removing CCB from antihypertensives). In the fast gait speed group there were 6 patients who took CCB (37.50%), while the slow gait speed group consisted of 13 patients who took CCB (86.67%). It was shown that the proportion of patients who took CCB in the slow gait speed group was significantly higher than that in the fast gait speed group (*p* = 0.009). Furthermore, by comparing the gait speed for the CCB medication group and non-medication group, it was shown that the gait speed of the medication group was significantly slower than that of the non-medication group (Table 4).

**Table 4:** Comparison of the gait speed between non-medication and medication groups

	Gait speed (m/s)				*p* value
	Non-medication group		Medication group		
CCB	1.15 [0.76–1.54]	*n* = 12	0.93 [0.70–1.40]	*n* = 19	0.020
Other antihypertensive	0.87 [0.75–1.54]	*n* = 8	1.12 [0.70–1.51]	*n* = 23	0.132
Other vasodilators	1.00 [0.70–1.54]	*n* = 28	1.12 [0.85–1.16]	n = 3	0.925

Data are expressed as median [minimum-maximum]

*p* value based upon Mann Whitney U test

In addition, the CCB taken by each patient were grouped based on the specific ingredients. Amlodipine, nifedipine, benidipine, cilnidipine were 16, 2, 1, and 1, respectively.

### Heat map analysis

**Fig. 2:**
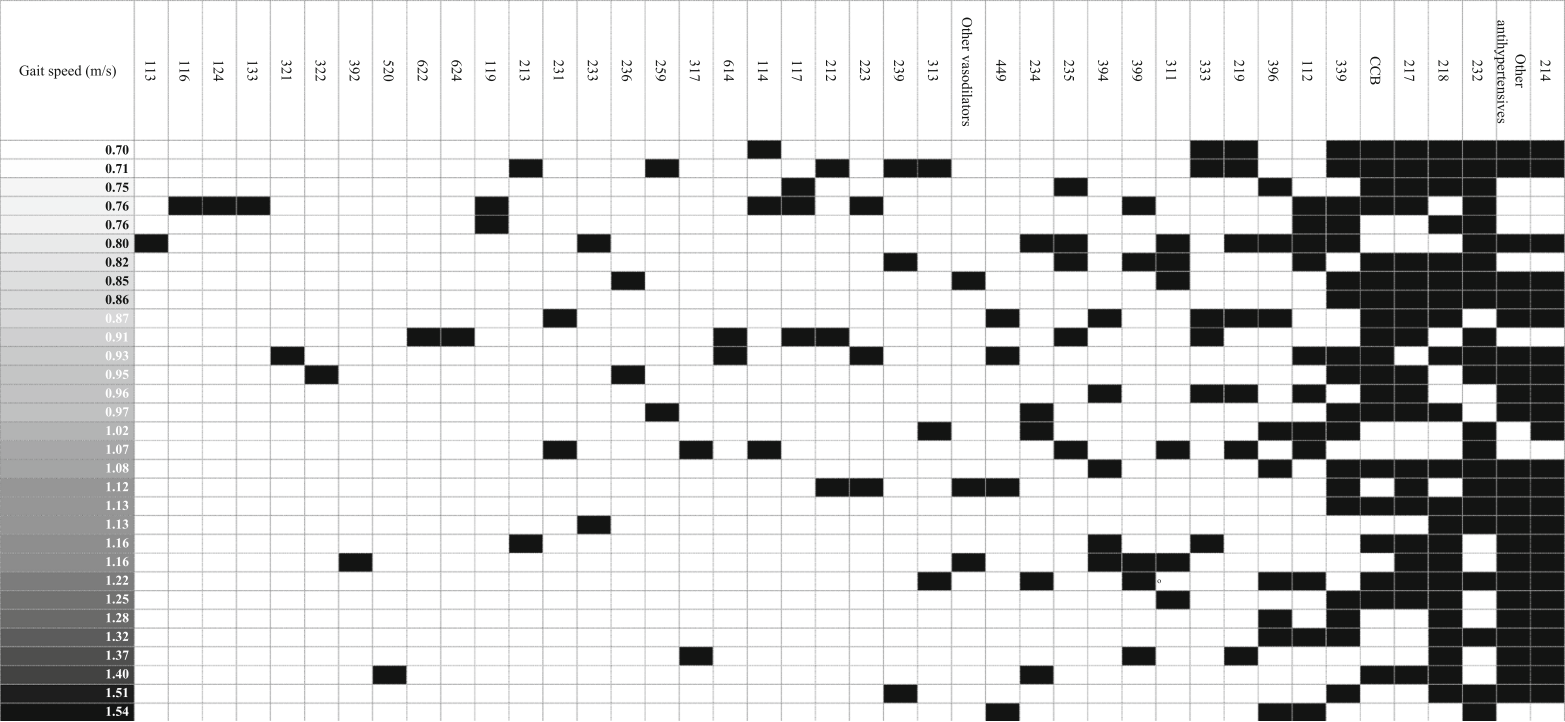
Patients were rearranged in order of gait speed, cells with the therapeutic category number corresponding to the medication being taken are shown in black. The lower the row the higher the gait speed

For this study, we focused on 6 or more patients, which was determined to be the average number of patients for each category. When examining the gait speed for the 217.Vasodilators, 232.Agents for peptic ulcer, and 333.Anticoagulants, a tendency was observed that the higher was the decrease in gait speed, the more patients took the medications (Fig. 2).

## Discussion

The results of the statistical analysis carried out in this study showed that a correlation existed between a decreased gait speed and increased number of medications. Subsequently, owing to careful examination and analysis of the prescription contents, it was observed that patients taking CCB had a decreased gait speed. In large-scale clinical trials or guidelines for the management of hypertension worldwide, CCB is regarded as the first choice for hypertension treatments owing to its excellent antihypertensive effect and safety profile [16–21]. In particular, this drug is widely used because it displays an excellent organ blood flow retention effect and is suitable for cases of organ dysfunction in the elderly [22].

In order to determine the factors associating the use of CCB with the gait speed, the effect of edema was at first assessed as a common adverse drug reaction of CCB [23]. CCB-induced edema is considered to be caused by fluid extravasation due to the fact that the vasodilatation action of CCB is larger in the peripheral arteries than in the veins, thus the arterioles expand without vasodilation of the venules, and the capillary pressure rises [24]. The edema may lead to a reduced range of motion [25, 26], as a result of a decrease in the gait speed. This correlates with the patient’s complaints of edema.

Experiments that subjected mouse soleus and extensor digitorum longus muscles to continuous stimulation in vitro under Ca^2+^-free conditions caused a dramatic increase of fatigue in the muscles [27]. CCB are medications used for the treatment of chronic diseases such as hypertension and cardiovascular diseases, and are commonly taken over a long period of time. By blocking for a long period of time the voltage-dependent calcium channels, which are one of the Ca^2+^ influx pathways to the skeletal muscle, it was assumed that the muscular fatigue would affect the muscle contraction, resulting in a decreased gait speed.

These findings are in agreement with a previous report that polypharmacy significantly increases the risk of frailty in older Japanese adults [28]. A similar study demonstrated that compared with participants taking 0–3 medications, the incidence of frailty was approximately double in those taking 4–6 medications and six times higher in people taking ≥7 medications during an eight-year follow-up [29]. On the other hand, other studies in healthy adults determined that the gait speed is associated with age, height, and lower limb muscle strength [30, 31]. Our results, however, indicate an association only between the gait speed and number of medications, most likely due to the differences in the patient characteristics. The participants to the reported study were in fact adults over 20 years, while the participants in our study were older outpatients over 65 years. In addition, no association was observed between the gait speed and stratum corneum moisture content. There have been reports suggesting that the stratum corneum moisture content and frailty maybe related [14], however their relationship is still unclear and additional work is required for acquiring a better understanding.

Hackett et al. reported that the risk of dementia is elevated in elderly over 60 years with a decreased gait speed [32]. In the present study, no participants were taking medications for dementia. Furthermore, upon confirmation the diagnosis disease to attending physicians, no participant with dementia. Thus, the association between the gait speed and dementia was not considered. Although dementia is the main condition requiring support or long-term care [13] in the elderly, focusing on the gait speed is deemed important to observe its further increase.

In the heat map analysis of the gait speed in the case of 217.Vasodilators, 232.Agents for peptic ulcer, and 333.Anticoagulants, we visually observed a higher tendency for patients taking these medications of having an increasingly slower gait speed. Some of the patients who took CCB and anticoagulants were affected by cardiovascular diseases. In patients with a cardiovascular disease, the risk of frailty increased from 2.7 to 4.1; it has been reported that the risk of becoming frailty in the follow-up period of three or more years increased 1.5-fold even if frailty did not exist at the baseline [33]. Thus, some patients who took CCB and anticoagulants were considered affected by frailty owing to the effect of the cardiovascular diseases, which led to a decrease in the gait speed.

The present study has some limitations that need to be considered. First, it was difficult to exclude the potential effects of unknown confounders other than those employed in the present study. Second, patients with dementia such as the Alzheimer’s disease were not included in the study as we targeted patients who were able to visit the pharmacy on their own, while patients with Parkinson’s disease did not participate since patients with gait disturbance were excluded. Another limitation is that the multivariate logistic regression analysis of height, weight, and BMI could not be carried out due to a lack of freedom. However, no correlation was found with the gait speed for any of the variables, therefore it was assumed that no relationship occurred with the gait speed. Therefore, to validate our result, a controlled prospective observational study will be required. Recently, there have been many reports about polypharmacy in Japan, and Kojima et al. described that the number of adverse drug reactions increases with an increased number of medications [4, 5]. In this study, we demonstrated that not only the number of medications should be considered as risk factor but also the prescription contents of the medications taken by older outpatients.

## Conclusions

In older outpatients with a decreasing gait speed, a high rate of CCB intake was observed. It was suggested that not only the number of medications but also the prescription contents could serve as an index to identify patients with a high risk of requiring support or long-term care.

Adverse drug reactions such as edema that can affect the gait speed may be developed upon CCB intake. Furthermore, in patients with edema, the risk of falling increases due to a decrease in the gait speed. By performing a proper prescription proposal, it can be assumed that the pharmacists may contribute to the reduction of the risk of needing support or long-term care required by the elderly.

## Data Availability

The datasets during and/or analysed during the current study available from the corresponding author on reasonable request.

## Abbreviations

a.u: Arbitrary unit
BMI: Body mass index
CCB: Calcium channel blocker
CI: Confidence interval
OR: Odds ratio
